# Minimally Invasive Approaches in Pediatric Urolithiasis. The Experience of Two Italian Centers of Pediatric Surgery

**DOI:** 10.3389/fped.2020.00377

**Published:** 2020-07-24

**Authors:** Francesca Destro, Giorgio Giuseppe Orlando Selvaggio, Mario Lima, Giovanna Riccipetitoni, Catherine Klersy, Neil Di Salvo, Federica Marinoni, Valeria Calcaterra, Gloria Pelizzo

**Affiliations:** ^1^Pediatric Surgery Unit, V. Buzzi Children's Hospital, Milan, Italy; ^2^Pediatric Surgery Unit, S. Orsola Hospital, University of Bologna, Bologna, Italy; ^3^Pediatric Surgery Unit, Fond. IRCCS Policlinico S. Matteo, Pavia, Italy; ^4^Clinical Epidemiology & Biometry, IRCCS Policlinico San Matteo Foundation, Pavia, Italy; ^5^Pediatric and Adolescent Unit, Department of Internal Medicine, University of Pavia, Pavia, Italy; ^6^Pediatric Unit, V. Buzzi Children's Hospital, Milan, Italy; ^7^Department of Biomedical and Clinical Science L. Sacco, University of Milan, Milan, Italy

**Keywords:** pediatric urolithiasis, urinary stones, endourology, minimally invasive, children

## Abstract

**Background:** Over the last 30 years, the incidence of pediatric urolithiasis (PU) has been increasing and the surgical management has evolved toward a minimally invasive approach (MIA). We reported the experience of two Centers of Pediatric Surgery in the management of PU, focusing on MIA as first choice in treatment.

**Methods:** Data were retrospectively analyzed from October 2009 to October 2019 in children with urolithiasis who were admitted to two referral Italian Centers of Pediatric Surgery. Demographic and clinical data of the patients, features of the urolithiasis, type of surgery were considered.

**Results:** Seventy patients (7.3 ± 5.0 years) with normal renal function were treated for calculi in the pyelocaliceal system (45.7%), ureter (34.3%), bladder (4.3%), urethra (1.4%), and multiple locations (14.3%). Size of calculi was >10 mm in 55.7% of cases (kidney>bladder/urethra>multiple>ureter, *p* = 0.01). Symptoms were present in 75.7% of patients. Family history was positive in 16.9% of cases. MIA was performed in 59 patients (84.3%): 11.8% shockwave lithotripsy (kidney>ureter>multiple); 32.2% ureteral retrograde surgery (ureteral>other localizations); 30.5% retrograde intrarenal surgery (kidney>other localizations); and 25.4% other procedures including percutaneous nephrolithotomy, cystoscopic bladder stone removal or laser cystolithotripsy (kidney>bladder>multiple). Preoperative stenting was necessary in 52.8% of cases. Four MIA procedures (6.9%, kidney>ureter/multiple) were converted to open surgery. Open surgery was required as first approach in 15.7% of patients (kidney>ureter>multiple) who needed urgent surgery or had associated congenital renal anomalies. In 18/70 of children (25.7%), with prevalence of stones in kidney and multiple location (*p* < 0.01), a second procedure completed the treatment (88.8% MIA). Intraoperative difficulties were recorded in 8.5% of cases, without difference between location and size of calculi. Late complications (5.7%) were related to displacement and infection of the ureteral stent.

**Conclusions:** MIA resulted to be feasible in more than 75% of primary surgery and in more than 85% of cases requiring a second procedure. Preoperative stent was mandatory in more than 50% of children. The technological evolution allowed to overcome many of the technical difficulties related to the approach to the papilla and lower calyxes. Open surgery is reserved for selected cases and endoscopic surgery represents the best choice of treatment for PU.

## Introduction

The incidence of pediatric urolithiasis (PU) is 1–2% of the adult population, but is progressively increasing (1–5). The familial predisposition and environmental factors, such as dietary practices as well as local climate characteristics, play a crucial role in stone formation in children (5, 6). Metabolic factors, including hypercalciuria, hypocitraturia, hyperuricosuria, and hyperoxaluria, are more common in PU than in adult stone disease. The urinary tract infections and genitourinary anatomical abnormalities, such as ureteropelvic junction obstruction and vesicoureteric reflux, represent additional and peculiar risk factors in PU (6–8).

The surgical approach to PU has radically changed over the last 30 years due to the widespread use of endoscopic and minimally-invasive approaches that are nowadays used as standard treatments (9, 10). The minimally invasive procedures are safe and more effective than open techniques (10–14). The use of dedicated pediatric instrumentation reduces risks of complications and induces some changes that we have observed in the disease itself (4, 15).

The aim of this paper is to report the 10-year experience of two Centers of Pediatric Surgery in the management of urolithiasis in children, focusing on minimally invasive approach (MIA) indication as first choice in treatment.

## Materials and Methods

### Patients

Data were retrospectively analyzed from October 2009 to October 2019, in pediatric patients (≤18 years) who were admitted to two referral Centers of Pediatric Surgery (Milan, Bologna, Italy) with a diagnosis of urolithiasis and an indication for surgery (symptomatic patients, calculi > 5–10 mm, depending on age and non-responders to medical treatment).

Demographic and clinical data were considered in all patients. Additionally, the features of the urolithiasis were also recorded.

The study protocol was approved by the Institutional Review Board of centers. The study was conducted in accordance with the 1975 Helsinki Declaration, as revised in 2008. All participants and/or their responsible guardians gave their written consent after being informed about the nature of the study.

## Methods

### Data Collection

#### Clinical Data

Clinical data included: age, gender, presence of associated anomalies or pathologies, use of drugs, symptoms at the diagnosis.

In all patients, we recorded the presence of pathological values of hemoglobin, markers of renal function, urinalysis parameters and PTH levels pre and post-operative (no single values were considered due to difference in range values during the years).

#### Features of the Urolithiasis

Features of the urolithiasis included etiological aspects, number and location of calculi, and diagnostic methods.

Diagnosis was confirmed with abdominal ultrasounds and x-ray in all cases. Patients with metabolic disease, congenital malformations, neurologically impaired children underwent evaluation with uro-CT. Uro-CT was also required in case of suspected lower ureteric or radiolucent stones that could have been missed with x-ray and ultrasounds. A total of 36 uro-TC (51.4%) were performed.

### Surgical Management and Monitoring

Type of surgical approach and outcome (as renal function and stone free rate at a minimum of 6 months post-treatment) were considered.

The management of patients treated with minimally invasive surgery included a multidisciplinary approach involving the evaluation by the pediatric surgeon with urological expertise and by the pediatric-nephrologist and the genetic study in selected cases.

The minimally invasive approaches used were: extracorporeal shockwave lithotripsy (ESWL), ureteral retrograde surgery (URS), retrograde intrarenal surgery (RIRS), percutaneous nephrolithotomy (PCNL), and other procedures such as stenting, cystoscopic bladder stone removal or laser cystolithotripsy. All the procedures were carried out under general anesthesia.

The open primary approach was adopted for patients who needed urgent surgery or had associated congenital renal anomalies requiring anatomical repair of the malformation combined to stone removal, together with complex history and previous multiple abdominal surgeries.

### Minimally Invasive Approaches

ESWL acts by a shock wave that gets inside the body and hits the calculus with minimal energy loss. Moreover, the new generation instrumentation uses a smaller focal area and provide less energy (14–21 kV with 1800–2000 shockwaves, up to 4000) with reduced risk of traumatism but the need for multiple sessions. This technique is not strictly a “minimally-invasive approach” but rather a “non-invasive approach”. However, in children, the procedure is performed under general anesthesia and in younger patients we always associate it to the insertion of a ureteral stent for the high risk of Steinstrasse, thus we considered ESWL as MIA.

In URS the patient was placed in a lithotomy position. The ureter was cannulated under fluoroscopic control during cystoscopy (9.5, 11, 14 ch cystoscope); a guide wire was left until the end and the procedure was performed under direct vision. The ureteral papilla was dilated with 8–10 fr dilators if the ureteronephroscope did not proceed easily (7.3 rigid, 7.5 flexible ureteronephroscope). RIRS exploits almost the same steps as URS but it permits an intrarenal surgery.

In URS AND RIRS when the retrograde passage of the instrument through the papilla was easygoing, the scope was inserted inside the ureter; in RIRS usually protected by a sheath (9.5 ch), and pushed up to the kidney, under direct vision and continuous washing. The tip of the laser fiber (Calculase System™, 230–365 μm fiber) was then inserted and it was used to hold the calculus in place, locking it against the calyx wall. The holmium:yttrium-aluminum-garnet (Ho:YAG) laser was generally set with 0.5–1.2 J of power and 5–15 Hz.

PCNL was performed with the patient in the supine or Valdivia position (the prone position is less used due to possible anesthesiologic difficulties and to the need for a concomitant transurethral access) (16). The procedure was preceded by a cystoscopy and ureteral stenting for retrograde pyelography. Intraoperative US was used to establish the exact entry site and the fluoroscopic control was performed to check the exact position. A J-tip guidewire was inserted through the inside of the needle that reached the desired calix. Amplatz dilatation set were then used to create the tunnel in which a protection sheath was inserted. The maximum dilation was obtained by an 18 ch Amplatz. A described alternative includes the use of balloon dilators but it determines a 30 fr size access therefore questioning the definition of MIA. Mini and ultramini-PNL was proposed as others MIA (12-13-14 fr sheaths) and recently, micro-PCNL approach was developed (4.85 fr “all-seeing needle”) (16, 17). The nephroscopes (12 ch) were used for endoscopic image and stones were directly removed by a grasper or broken by lithotripsy.

During cystoscopic bladder stone removal, the stone was passed through the urethra after cystoscopic anatomical evaluation and definition of stone burdens. In case of stone not small enough, cystolithotripsy was performed. Fragments were then easily washed out or removed with retrieval devices. Cystoscopy also provided for the insertion of a ureteral stent through the ureteral papilla. The double J (DJ) stent was advanced to the kidney and kept in place by the two coils at its ends.

A preoperative DJ stenting (*n* = 37) was inserted for 1–3 weeks in all cases presenting with narrow ureteric lumen to allow the passive ureteral dilatation and to provide an safer ureteral insertion of devices during surgery, considering the dimension of the papilla.

A postoperative DJ stenting (*n* = 49) was used to prevent ureteral obstruction by residual fragments, bleeding, and edema.

### Statistical Analysis

Qualitative variables were described as counts and percentages. Quantitative variables were expressed as the mean value and standard deviation. Statistical analyses were performed using the exact Fisher test for comparison of categorical variables and the Kruskall Wallis (for more than 2 groups) or the Mann-Whitney *U*-test for continuous variables. All tests were two-sided and a *p*-value below 0.05 was considered statistically significant.

The data analysis was performed with the STATA statistical package (release 16.1, Stata Corporation, College Station, Texas, USA).

## Results

### Clinical Data

Seventy patients (mean age 7.3 ± 5.0 years; range age 7 months-18 years; 22F/48M) were treated for calculi. Symptoms were present in 75.7% of cases, in particular the patients showed one or more symptoms, including abdominal pain, urinary tract infection, hematuria or an association of them in 32.9, 24.3, 12.8, and 5.7% of cases, respectively. All patients presented with a normal renal function. Other laboratory tests were normal, excluding hypercalciuria in 9 patients and hypocitraturia in 6 cases.

As reported in Table 1, in 36/70 of cases (51.4%) the calculi were idiopathic; in the remain subjects pathogenic factors were supposed, including congenital or acquired anomalies (20/70, 28.6%), neurological bladder dysfunction or previous urologic surgery (11/70, 15.7%), metabolic diseases (2/70, 2.8%), and drugs assumption (1/70, 1.4%). Family history was positive in 16.9% of cases.

**Table 1:** Clinical data and features of the urolithiasis in the enrolled patients.

Number of patients	70
Age at treatment	
Mean SD (yrs)	7.3 ± 5.0
Gender (M/F)	48/22
Cause (*n*, %)	
Congenital anomalies and anatomical alterations^*^	20 (28.6%)
Functional dysphunctions^**^	9 (12.8%)
Previous urological surgery (e.g., bladder-augmentation)	2 (2.8%)
Metabolic disease	2 (2.8%)
Drugs	1 (1.4%)
Idiopathic	36 (51.4%)
Symptoms (*n*, %)	53 (75.7%)
Abdominal pain	23 (32.9%)
Urinary tract infection	17 (24.3%)
Hematuria	9 (12.8%)
Multiple symptoms	4 (5.7%)
No symptoms (*n*, %)	17 (24.3%)
Side (right/left)	26/44
Treatment	
Single	53 (75.7%)
Multiple	18 (25.7%)
Location	
Bladder	3 (4.3%)
Kidney	32 (45.7%)
Ureter	24 (34.3%)
Urethra	1 (1.4%)
Multiple	10 (14.3%)

* *Pyelo-ureteral junction obstruction, obstructive megaureter, horseshoe kidney, vescico-ureteral reflux and bladder pathologies (exstrophy, epispadias)*.

** *Neurologic bladder, myelomeningocele*.

### Features of the Urolithiasis

Size of calculi was >10 mm in 39/55 (55.7%) of cases (mean diameter 12.5 ± 5.4 mm; 51.2% kidney, 23.1% urether; multiple locations 18%, other sites 7.7%). An association between presence of symptoms and diameter >10 mm was noted (*p* = 0.01; 64.1% in size >10 mm vs. 90.3% ≤ 10 mm). No correlation between size >10 mm and age and sex of the patients was recorded (*p* = 0.09 and *p* = 1, respectively).

Calculi were localized in the pyelocaliceal system in the 45.7% of cases, in the ureter in 34.3% of patients, in the bladder and the urethra in 4.3 and 1.4% of children respectively; multiple locations were observed in the 14.3%.

No significant difference in localization was noted according to sex (*p* = 0.6) and familiar predisposition (*p* = 0.7).

In children with neurological bladder dysfunction a higher prevalence of the calculi in the kidney was noted compared to others locations (*p* = 0.04). No other significant associations were recorded between pathogenic mechanism (e.g., use of drugs, history of previous urological surgery, presence of congenital malformations, and metabolic disorders) or the onset of symptoms and site.

The diameter of the calculi in the kidney 14.3 ± 6.4 mm was higher compared to other localization bladder/urethra 14.2 ± 6.2 mm, multiple locations 12.2 ± 2.8 mm, ureter 9.8 ± 3.1 (*p* = 0.01).

Patients with kidney or bladder stones were older compared to subjects with other localizations of calculi (14.3 ± 6.4 vs. 10.9 ± 3.7 yrs, *p* = 0.02 and 17.3 ± 2.5 vs. 12.2 ± 5.4 yrs, *p* = 0.04, respectively); on the contrary the subjects with ureteric stones were younger compared to other patients (9.8 ± 3.1 vs. 13.8 ± 5.8 yrs, *p* = 0.001).

### Surgical Management and Monitoring

MIA was performed in 59/70 patients (84.3%, 21F/38M, mean age 7.7 ± 5.0 years), in particular, ESWL in 7/59 of cases (11.8%), URS in 19/59 of subjects (32.2%), RIRS in 18/59 (30.5%), other procedures, such as PCNL, stenting, and cystoscopic bladder stone removal or laser cystolithotripsy, in 15/59 of children (25.4%). Preoperative stenting was necessary in 37/59 cases (52.8%) with narrow ureteric lumen (*n* = 18) and pus coming out of the ureter (*n* = 7). In 12 cases, the stent was used as a bridge treatment for symptoms requiring an urgent approach. Age and sex distribution into surgical group was not significantly different (*p* > 0.5).

Significant difference in position of stones was noted according to type of surgery (*p* = 0.001). In particular ESWL was predominantly performed for treatment of the kidney stones (71.4%) compared to ureter and multiples locations (14.2%); URS was almost exclusively indicated for ureteral stones (73.7%), and less frequently for other localizations (26.3%); RIRS was used for kidney stones (66.7%) and less frequently in other localizations (33.3%); other procedures were considered respectively for renal (47.1%) and bladder localization (23.5%) and/or multiple localizations (29.4%).

Postoperative stenting was performed for treatment of pyelocalical system (45.0%), ureter (36.7%), or multiple locations (18.3%).

Four MIA procedures (4/59, 6.89%) for treatment of kidney and/or ureter/multiple large stones were converted to open surgery. In particular, conversions were required in two patients younger than 3 years (one of them with a renal calculus, the other with a ureteral stones), in one patient with neurogenic bladder and solitary kidney and in one syndromic patient with a large bladder stone, treated at the beginning of our experience with an energy device that was scarcely effective in fragmenting the calculus.

An association between diameter >10 mm and type of surgery was noted (*p* = 0.02), with predominant association with RIRS.

Open surgery was required as first approach in 11 patients (11/70, 15.7%, 1F/10M, mean age 7.2 ± 4.9 years) who needed urgent surgery (*n* = 3) or required an anatomical correction of the urinary tract (complex cases submitted to pyeloplasty for pyelo-ureteral junction obstruction, ureteral reimplantation for obstructive megaureter and bladder augmentation in ex exstrophy-epispadias patients) combined to stone removal (*n* = 8).

In 18/70 of children (25.7%), with prevalent location of stones in kidney and multiple sites (*p* < 0.01); in 16/18 cases (idiopathic urolithiasis in 10 cases) a second procedure completed the treatment (16/18, 88.9% MIA and 2/18, 11.1% open surgery). Patients requiring multiple procedures showed an idiopathic urolithiasis in 10 cases and the remaining 8 patients (6 approached by MIA) had an association with SMA, metabolic disorders, pyelo-ureteral junction obstruction or neurologic bladder.

The average hospital stay was 2.3 days (range, 12 h to 5 days); the mean follow-up was 4 years (range, 1 month to 11 years).

Intraoperative difficulties were recorded in 6/70 (8.5%) of MIA cases due to the blurred vision of the papilla, pus coming out from the ureter and difficult access to the lower calyxes. No difference between location and size of calculi were recorded (*p* > 0.5). Hemoglobin remained stable without the need for transfusion. Postoperatively, complications rate as per Modified Clavien Dindo classification was 12.8%: 5 patients were kept longer for analgesic and antipyretic therapy (grade I); displacement and infection of the DJ stent were registered in 5.7% of the cases (4 patients) and required stent removal under general anesthesia (grade IIIb).

## Discussion

Pediatric urolithiasis is a urinary system disease encountered in clinical practice more and more frequently. Medical treatment, as the first line treatment, is effective only in minority of the cases. Surgical approach is estimated to be necessary in 22 to 60% of children with nephrolithiasis (18).

The management of pediatric patients with urolithiasis is very complex and poses a challenge for the Pediatric surgeon and urologist. The appropriate surgical therapy depends on localization, diameter of calculi, as well as on the anatomy of the urinary tract (14, 19, 20), associated urogenital malformations in children and size of pediatric patients.

The standard MIA to treat PU do not differ from those used for adults and includes ESWL, URS, RIRS, PCNL, and preoperative stenting (14, 21). The development of dedicated miniaturized instrumentation and the widespread use of minimally invasive techniques in children has been fundamental for the pediatric surgical management of urolithiasis (6, 11, 12, 22, 23).

Failure to access the ureter through the papilla has been indicated as a common reason of treatment failure in children (11). In our series, technical difficulties (mostly impossibility to overcome the papilla but also to reach renal stones) were the main reasons for conversion to open surgery. More than 8% of adult patients present difficulties to tight the ureter and more than 10% of them require preoperative DJ stenting (24). In our experience preoperative DJ stenting was required in almost 50% of the patients. Preoperative stenting overcomes a problem, although it exposes children to an additional anesthetic procedure. Other options to access the ureter including the ureteral dilatation and alfa-blockade are experienced in adults but only a few cases of ureteral dilatation are reported in pediatrics (25, 26). The reasons for the high percentage of pre-stenting in our series are also attributable to cases with concomitant urinary infection and pus coming out from the papilla and, at the beginning of our experience, the need for urgent surgery when the instrumentation was not immediately available.

URS is indicated in patients with distal ureteral stones and its complication rate is reported to be associated to the duration of the procedure (14); in this study RIRS resulted to be more useful for medium sized kidney stones (>10 mm) but this data is probably reflecting a personal experience. We showed that ESWL has been the preferred option for small renal stones or proximal ureteral calculi (21). We usually reserve PCNL for patients with huge renal stones (e.g., complex Staghorn calculi, > 20 mm) or for secondary treatment.

Additionally, as reported in this study, clinical data of the patients, including age and associated pathologies, such as bladder dysfunctions, influence the features of stones and may also play an important role in the surgical decision to obtain the best surgical outcomes. In the same way, the features of the stone in terms of composition, influence the therapeutic success. For these reasons, preoperative clinical and radiological assessment helps in predicting the outcomes. Low-dose CT scan with determination of Hounsfield unit gives information regarding the stone nature (e.g., soft infective stones versus metabolic hard cysteine stones), together with an efficacy in stone finding that is around 97%, but the technique carries well-known potential radiation hazards (data from our radiological department shows that single abdominal x-ray provides 0.02 mSv while low-dose abdominal CT without contrast means provides 0.5 mSv).

MIA resulted to be feasible in more than 75% of primary surgery and in more than 85% of cases requiring a second procedure, confirming that it may represent the best choice of treatment also when multiple treatments are required. Complication rate in our series was 12.8% (only 5.7% if we exclude grade I complications according to Modified Clavien Dindo), indicating that MIA is safe in more than 87% of cases. Technological evolution has allowed to face many technical difficulties, including those related to the anatomy of the ureter, the papilla and the access to the renal lower pole. A combination of different treatment methods should be taken in consideration to complete the treatment and to achieve optimal stone-free rates (27–30).

As in our patients, open surgery is reserved for selected cases, especially those complex patients, with a history of multiple surgical procedures, who need for anatomical correction of the urinary tract combined to stone removal.

As reported in the literature, the advantages of MIA over ‘open’ surgery, including faster recovery times, less pain and discomfort, reduced surgical invasiveness, are obvious and unequivocal (9, 14, 20, 31). In male patients this approach additionally protects the urethra; this is more relevant in those patients with chronic vesical problems that may induce further vesical stone formation and consequently more stone removal procedures (31).

To ameliorate the minimally invasive treatment of the urolithiasis, as suggested by Silay et al., future efforts will have to be addressed to optimize instruments size for the adaptation in children (11). The optimization process is intended not only as the reduction in the diameter of our tools but also as adjustments in length and improvement of the maneuverability of the instruments when needed. Additionally, a better patient selection translates into higher success rates and improved safety; to accomplish this task first attempts have been made to combinate artificial intelligence and new technological developments in the field of endourology in adults (32).

The improvement of the energy sources and the promotion of the use of high-power lasers may help to reduce operating times and the risk of complications related to long and multiple procedures in the treatment of large, high-density stones (e.g., cystine) (22). However, precautions should be taken into consideration due to fragmentation, dusting, popcornig phenomenon with stone retropulsion and intrarenal temperature elevation (33). Efforts have been made to combine high-power lasers with specific ways to deliver energy (e.g., pulse modulation Moses technology) to increase the safety profile (33). Furthermore, the propose of technical improvements, such as changes in patient position during PNL (supine approach) and the promotion of other MIA robotic surgery will improve the quality of care (11).

We are aware that there are some limitations in our study, and the retrospective design has numerous disadvantages with an inferior level of evidence. Secondly, we considered a limited sample size; therefore, a larger cohort of patients is mandatory to ameliorate the analysis of the results, also considering a high volume centers. Additionally, the lack of clinical data (e.g., BMI) may have limited the interpretation of pathogenesis of stones and this could represent possible factors influencing stone features and choice of surgery.

In conclusion, MIA resulted to be feasible in more than 75% of primary surgery and in more than 85% of cases requiring a second procedure. Preoperative stent was mandatory in more than 50% of children. Localization and size of stones, clinical data, and the anatomy of the urinary tract play a crucial role in the surgical decision and outcome. The place of open surgery is reserved for very selected cases and endoscopic surgery represents the best choice of treatment for PU.

## Data Availability Statement

All datasets generated for this study are included in the article/ supplementary material.

## Ethics Statement

The studies involving human participants were reviewed and approved by Ospedale dei Bambini Vittore Buzzi, Milano. Written informed consent to participate in this study was provided by the participants' legal guardian/next of kin.

## Author Contributions

FD contributed conception and design of the study, recorded and organized the database, and wrote the first draft of the manuscript. GS performed the surgical interventions and recorded the data and supervised the manuscript. ML and GR contributed conception and design of the study and performed the surgical interventions. CK organized the database and performed the statistical analysis. ND and FM contributed conception and design of the study and recorded the database. VC organized the database and wrote and supervised the manuscript. GP contributed conception and design of the study and wrote and supervised the manuscript. All authors contributed to the article and approved the submitted version.

## Conflict of Interest

The authors declare that the research was conducted in the absence of any commercial or financial relationships that could be construed as a potential conflict of interest.
